# How the Media Places Responsibility for the COVID-19 Pandemic—An Australian Media Analysis

**DOI:** 10.3389/fpubh.2020.00483

**Published:** 2020-08-21

**Authors:** Trevor Thomas, Annabelle Wilson, Emma Tonkin, Emma R. Miller, Paul R. Ward

**Affiliations:** College of Medicine and Public Health, Flinders University, Adelaide, SA, Australia

**Keywords:** media analysis, responsibility, COVID-19, framing analysis, thematic analysis, blame

## Abstract

Global pandemics are likely to increase in frequency and severity, and media communication of key messages represents an important mediator of the behavior of individuals in response to public health countermeasures. Where the media places responsibility during a pandemic is therefore important to study as blame is commonly used as a tool to influence public behavior but can also lead to the subjective persecution of groups. The aim of this paper is to investigate where the media places responsibility for COVID-19 in Australia. Specifically, we identify the key themes and frames that are present and observe how they changed over the course of the COVID-19 pandemic in relation to government actions and progression of the pandemic. Understanding media representations of the COVID-19 pandemic will provide insights into ways in which responsibility is framed in relation to health action. Newspaper articles from the Australian and the Sydney Morning Herald were sampled between January 20 and March 31 2020 on every second Monday. Factiva was used to identify and download newspaper articles using the following search criteria: “COVID-19” OR coronavirus OR “Wuhan virus” OR “corona virus” OR “Hebei virus” OR “wet market” OR (Wuhan AND virus) OR (market AND Wuhan and virus) or (China AND Virus) or (Novel AND Virus). Articles were imported into Nvivo and thematic and framing analyses were used. The results show that framing of the pandemic was largely based on societal issues with the theme of economic disruption prevalent throughout the study time period. Moral evaluations of the pandemic were infrequent initially but increased co-incident with the first signs of “flattening of the curve.” Explicit examples of blame were very rare but were commonly implied based on the causal origin of the virus. The Australian printed media were slow to report on the COVID-19 pandemic, in addition they were reluctant to apportion blame until the end of the study period, after confirmed case rates had begun to slow. This is interpreted as being due to an evaluation of the pandemic risks as low by the media and therefore the tools of othering and blame were not used until after the study period when the actual risks had begun to abate, more consistent with an inquiry than a mediating mechanism.

## Introduction

With global confirmed cases approaching 13.1 million and confirmed deaths approaching 574,000 (at July 2020) (1) the COVID-19 (SARS-CoV-2) pandemic of 2019–2020 represents the largest public health emergency since the Spanish flu of 1918 (2).

In Australia, newspaper and television information campaigns have been announced by the Prime Minister as a source of information for the public during the evolving COVID-19 pandemic (3). Through these various forms of media, the public health and economic response to the pandemic in Australia has been swift and advised by public health officials and epidemiologists (3). This was demonstrated by the activation of a COVID-19 emergency response plan by the federal government on 27 February 2020 (4) a pandemic preparedness response plan released in advance of the World Health Organization (WHO), which announced a COVID-19 public health emergency on 30 January 2020 (5) with an escalation to “pandemic” characterization on 11 March 2020 (6). To date, extensive public health guidelines, mandated by the Australian federal government, have been announced to limit person-to-person transmission within the public. Guidelines have been based on numerical modeling and the pathogenesis of the disease. Modeling has demonstrated that without 80–90% compliance by the public the pandemic could not have been controlled and that the early intervention by the federal government has meant that, to date, Australia has largely avoided the high mortality rates associated with the exponential rise of cases of COVID-19 relative to many other first-world nations such as the US, UK, and Italy (7, 8).

Reviews into biosafety suggest that epidemics not-unlike COVID-19 are likely to increase in frequency and become more harmful due to globalization and an increase in human-animal contact (9). Therefore, the media's timing and reporting of accurate statistics and advice represents an important topic for discussion with respect to public health emergencies. Previous work discussing mistrust of the media to provide accurate information to the public has shown the material effect of poor perception of the media and therefore poor public response to crises (10, 11).

Previous epidemics, such as Avian Flu and other threats of pandemic influenza, have led researchers to explore the media-driven messages portrayed to the public through newspapers (12–16). These examples serve to highlight the importance of media messaging, as the implications for non-compliance can have dire effects on public health. The ability to deduce personal risk and therefore compliance with government mandated guidelines is associated with trust of the media. Therefore, how the media portray health crises is an important influence not only on public behavior (17) but also on the long-term repercussions for health (18).

The framing of responsibility during health crises is known as a sense-making and coping mechanism for individuals, but which can also lead to stigmatization of an affected group (19). Therefore, the role of the media and how they frame responsibility (e.g., on individuals and/or institutions for their various roles and responsibilities) for a health crisis represents an important component of messaging to the public. By placing responsibility for a health crisis, such as a pandemic, the media are also able to mediate public behavior to panic by inducing a sense of otherness which has the effect of allaying fears by framing them as distant (20).

This study evaluates how two high readership and broad demographic newspaper media outlets frame responsibility for COVID-19. The study applied a qualitative approach to both framing and thematic analysis to the initial 11 weeks following the first publications of the COVID-19 pandemic by the two newspapers, the Australian and the Sydney Morning Herald. This study contributes to the limited literature on how the media have responded to the COVID-19 pandemic and was completed in response to calls for how the media portrays COVID-19 (18).

## Methods

### Scoping the Dataset

Newspapers selected for the media analysis were limited to Australian print media that were accessible through the electronic database Factiva. When planning the study, multiple newspapers were considered for inclusion in the media analysis (Search 1—Table 1). However, given the exceptionally high article numbers (*n* = 8,536) related to the search terms used to scope the dataset (Table 2), two newspapers were ultimately chosen to limit the sample size, The Australian (AUS) and the Sydney Morning Herald (SMH) (Search 2—Table 1). The rationale for selection of these two newspapers were that they offer high readership ADDIN EN.CITE (21, 22), have traditionally diverse political orientations (23, 24) and diverse readership demographics (25). Articles in both newspapers were limited to those that were printed, with the following excluded: online publications, blogs and audio and visual media. Printed newspapers are associated with higher credibility than their online counterparts (26, 27). Given the importance of credibility in the media related to past pandemics (10), we selected print media as the preferred media medium (10, 28, 29).

**Table 1:** Search method summary.

	**Newspapers**	**Time period**	**Article number**
Search 1	The Australian, The Advertiser, Sydney Morning Herald, The Age, Australian Financial Review	1 October 2019 to 31 March 2020 (past 6 months)	*n* = 8,536 articles total
Search 2	The Australian, Sydney Morning Herald	1 October 2019 to 31 March 2020 (past 6 months)	*n* = 3,878 articles total
Search 3	The Australian, Sydney Morning Herald	20 January to 31 March 2020	*n* = 3,868 articles total
Final Dataset	The Australian, Sydney Morning Herald	20 January 2020 to 31 March 2020 Every second Monday starting 20 January 2020 ending 30 March (6 days over 11 weeks)	*n* = 313 articles total (*n* = 171 The Australian; *n* = 142 Sydney Morning Herald)

**Table 2:** Search terms used to acquire article database.

Search terms	“COVID-19” OR coronavirus OR “Wuhan virus” OR “corona virus” OR “Hebei Virus” OR “wet market” OR (Wuhan AND virus) OR (market AND Wuhan AND virus) or (China AND virus) or (novel AND virus)
Search region	Limited to “Australia”

### Search Terms

The search terms used for the study aimed to capture all articles available within the respective newspapers as related to the emerging COVID-19 pandemic and time period thereafter. Search terms were selected based on the changing nomenclature of the SARS-CoV-2 virus and its assumed epidemiological provenance (Table 2).

### Time Period

**Figure 1:**
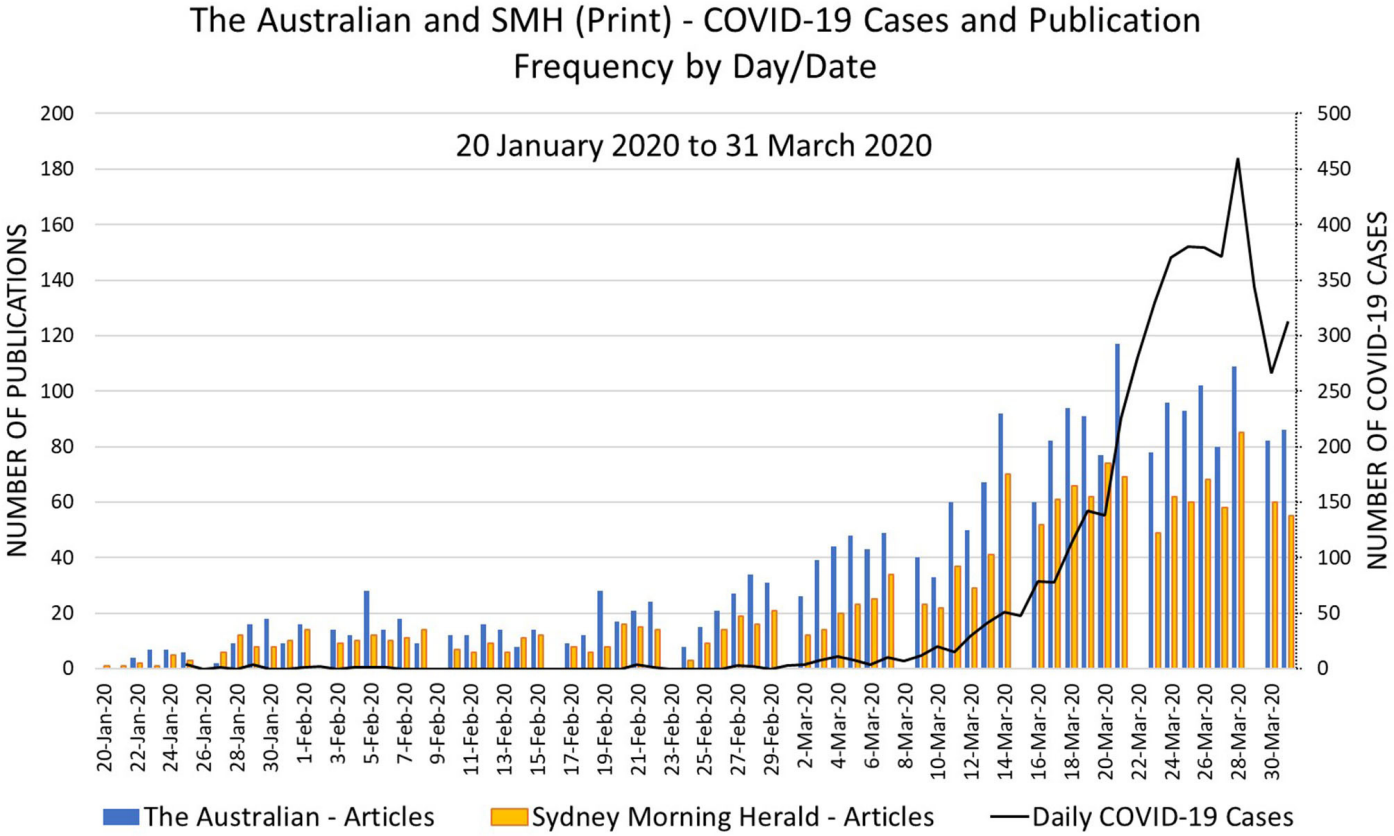
Publication and COVID-19 frequency per day for the study time period.

The selection of a time period for this study was based on the emergence of COVID-19 around late-November 2019 ADDIN EN.CITE (30, 31), until 31 March 2020 (date of commencement of this study) (see Figure 1). To manage the large number of articles while retaining an accurate representation of the publications, articles published fortnightly on a Monday were selected for inclusion. Mondays were selected as they generally represented the start dates of many government reforms. Additionally, Mondays typically (but not always) followed the major announcements by the Australian federal and state governments related to COVID-19 status updates and associated guidelines. This selection was informed by the view that coverage of public health emergencies is highly event based with publication frequency reflecting case numbers and government action (32). The rationale for the fortnightly selection component was based on reducing the number of articles for analysis from *n* = 670 every Monday to *n* = 313 every second Monday given timing constraints.

The included articles were copied from Factiva to Microsoft Word, split into individual articles, arranged by date and appropriately named according to their Newspaper and order of publication (e.g., AUS001, AUS002) before uploading to Nvivo 12 (QSR International, Doncaster) for analysis. Articles were excluded if their content only made passing or no reference to COVID-19.

### Analysis

To explore how the media place responsibility for COVID-19 we took an inductive thematic and framing analysis approach to media analysis as previously used by Foley et al. (33) This consisted of the hand-coding of separate thematic and framing analysis. Hand-coding as opposed to computer aided searches for characteristic search terms was selected as it allows for the comprehension of the sample articles beyond literal definitions (34).

Thematic analysis is a commonly used qualitative data analysis technique that seeks to identify common themes, ideas, and patterns within a given dataset (35). The technique was selected as it offers flexibility in application to an inductive approach to data analysis and its previous use in media analyses (34, 36, 37). Qualitative framing analysis is also commonly used in media analysis given its usefulness as a heuristic tool (33, 38). To complete the thematic analysis, author TT began thematic analysis of the articles chronologically starting with articles from the SMH followed by The Australian. Initially a selection of articles was read and major themes were noted to familiarize the author with the content. Axial and selective hand coding of the data followed in the NVivo software package to develop initial coding structures which were reviewed by Authors AW, ET, EM, and PW. This early stage coding later developed into more complex aggregations of nodes that were defined by their similarity. For example, nodes associated with the economic impact of COVID-19 that were loosely distributed within the coding “tree” were grouped under more definitive categories including “Disruption/Economic/Financial market” or “Education.” During thematic analysis, Author TT frequently presented and discussed observations, methods and updates of the analysis with Authors AW, ET, PW, and EM.

As outlined by Foley et al. (33, 39) we applied a framing analysis approach adapted from Entman (40), which was adapted by Matthes and Kohring (41). This framing analysis method defines the Social, Medical and Behavioral frames as each being made of four frame components Causal Attribution, Moral Evaluation, Problem Definition, and Treatment Recommendation. These frame components form logical divisions within each frame into which data can be appropriately coded or categorized (see **Tables 4**–**6**). As outlined by Foley et al., the framing analysis followed thematic analysis (39). However, unlike Foley et al., this was undertaken immediately after completion of every ten thematic analyses. The decision to change the methodology was driven by timing constraints and differed from the Foley et al. method which called for framing analysis to only begin after the completion of all thematic analysis (39). Conducting the framing analysis in small batches allowed for knowledge retention of the themes and narrative which enabled faster decision making for the framing analysis. Consistent with the thematic analysis process, observations and discussions of the framing analysis were communicated to Authors AW, ET, EM, and PW.

A final review of the data by Author TT queried the themes and words frequency of individual nodes as coded. A word search within the articles of those common terms presented a list of paragraphs where those terms appeared uncoded during the first pass analysis. These terms were checked for relevance to the thematic and framing analysis and either added or passed over. This final check yielded few additions to the analysis, confirming that the majority of the codes had been captured already.

Following the completion of both thematic and framing analysis, the database of results was reviewed by authors ET and AW for completeness and decision rationale. No significant changes to the analysis were made following the review.

## Results

A total of 313 articles were identified, 310 included, and three excluded. Articles were excluded if their content only made passing or no reference to COVID-19.

Following significant coding, both thematically and through framing analysis, it became apparent that substantial overlap of the themes and the framing existed. This was made evident on the completion of the coding of the SMH articles in their entirety and the aggregation of the thematic codes. For example, the hand-coded themes of “disruption” and their sub-categories of “financial markets, sport, schools etc.” were largely a reflection of the “societal” frame and “problem definition” frame component. Similarly, thematic coding of “opinions” and subcategories of “positive/negative” and child categories of “government, process, community” etc. were almost direct reflections of the “societal” frame and “moral evaluation” frame components. In large, the thematic coding represented an albeit more granular representation of the framing analysis. For this reason, the results are presented as the thematic analysis results as represented within the frames.

Quotes are referenced with their Newspaper ID and their frame and frame component categorization as per coding completed in Nvivo. For example: (SMH025)—Societal—Problem definition: “Example quotation” (Reference) would mean paper 025 from the SMH, in the societal frame and problem definition frame component.

### Results Summary

The societal frame was consistently the dominant frame throughout the analyzed time period. From the onset, reporting of COVID-19 was dominated by the disruption of business, education and sports as a result of its transmission and social distancing guidelines. These themes were presented in the problem definition frame component for the societal and medical frames predominantly. Over time, the frame components of treatment recommendations and moral evaluations became more prominent with moral evaluations only becoming particularly prevalent toward the final weeks co-incident with the first signs of “flattening the curve.” The causal attribution of the framing analysis did not change substantially during the time period and was often simply noted as “due to the spread of coronavirus.”

From this analysis, the factor which appeared to have the greatest impact on how the media framed the COVID-19 pandemic was time, therefore the findings are presented in three time periods as per Table 3 (1–Beginning, 2—Middle and 3—End). Each time period is presented as a framing analysis rubric which notes the common themes present in each frame and frame component. The matrices/rubrics are shaded to indicate the prevalence of themes within the frames, with darker shades indicating prominence. Following the methodology of Foley et al. decisions on the relative prevalence and therefore shade of the components were made by (39).

**Table 3:** Study time period divisions.

**Group**	**Time period**	**Articles**
1—Beginning	20/01/20 to 03/02/20 (Weeks 1 and 3)	24
2—Middle	17/02/20 to 02/03/20 (Weeks 5 and 7)	55
3—End	16/03/20 to 30/03/20 (Weeks 9 and 11)	234
Total		313

#### The Beginning (Weeks 1 and 3)

During the early stages of the pandemic, the main issues presented concerned the definition of the problem at-hand. The dominant themes focused on the imminent threat to the economy. At this stage the media was coming to terms with the epidemiological nature of COVID-19 as based on foreign data and what it might mean for the economy at large. The first reported case of COVID-19 in Australia was confirmed on 25 January 2020 and this stimulated a rise in reporting on the topic. Table 4 shows the frames and frame components most prominent in the early stages of the COVID-19 pandemic.

**Table 4:** Week 1 and 3, framing of COVID-19 (*n* = 24).

	**Causal attribution**	**Moral evaluation**	**Problem definition**	**Treatment recommendation**
Medical	Virus outbreak		Increasing cases Pneumonia, new coronavirus Origin Wuhan, China Symptoms and timing of, unknown	Flatten the curve Slow the spread Healthcare workers at borders—screening Seek medical advice if unwell Face masks and health checks Report people who show symptoms
Behavioral		Hypocritical actions Confusion, lack of information at airports Disappointment and helplessness due to poor process	Individuals separated from family	Calm down
Societal	Spread of coronavirus Pandemic High density living	Financial Public health at the expense of business Positive Gov. fast acting Gov. improved vs. bushfires	Economic impacts border closures supply lines disrupted Global financial market Unknown timing of impacts	Cancel flights Screening Travel advice Downgrade economic forecasts Evacuations Gov. imposed quarantine Control spread of Coronavirus Businesses call for aid

*Shading of cells represent relative frequency of themes (white = uncommon, light gray = few instances, dark gray = common, black = very frequent occurrence)*.

The first attempts to curtail the effects of COVID-19 included international border control. The immediate effect from this public health directive was the reduction of tourist numbers and restricting international students from entering the country. The net effect of these restrictions was highlighted in the articles primarily as financial disruptions to both the tourism and education sectors. These were coded as per the framing categories as falling within the “societal” frame and “problem definition” frame component. For example:

(AUS010)—Societal—Problem Definition: “Australian Tourism Industry Council executive director Simon Westaway said the sudden halt to Chinese visitors would have ramifications for much of the industry and steps would need to be taken to develop a recovery plan. “The Chinese market is a 1.5 million-visitor-a-year market for Australia, bigger than New Zealand, and it generates in excess of $12 bn in annual tourism receipts,” Mr. Westaway said.” (42).

(AUS011)—Societal—Problem Definition: “China is a critical economic partner for us. They”re the greatest source of foreign students—over 200,000 into Australia—(and) 1.4 million tourists,” Mr. Frydenberg said “Together, those two sectors provide about $16 bn to the Australian economy. And they are the recipients of around 30 per cent of our trade (43).”

The medical frame appeared as secondary to the societal frame. Themes present in the Medical Frame commonly included both domestic and foreign case numbers and potential symptoms. At this stage, data on the virology and timing of the symptom expression were sparse and resulted in broad treatment advice.

Moral evaluations were primarily limited to the Societal Frame and followed generally positive feedback toward the Government's response and negative sentiment toward the disruptive nature of COVID-19 related guidelines. Positive feedback of the Government was highlighted in contrast to the recent bushfires, an Australian natural disaster, where the government's response was commonly derided by the media for its poor performance. For example:

(AUS003)—Societal—Moral Evaluation: “Morrison [Prime Minister of Australia] has more than made amends for his missteps [bushfire disaster] and his rapid response to the potential pandemic posed by the coronavirus cannot be faulted. Morrison is now battling multiple national crises. Drought, fire, and plague. While foremost issues of human need and the well-being of the nation, there is an obvious political effect. And ultimately an economic one. Morrison has yet to shows signs of panic (44).”

(SMH005)—Societal-Moral Evaluation: “Some were angry at the government's decision to send them [Australians stranded in Wuhan, China] to Christmas Island [refugee detention center], while others said they were not being given enough time to evacuate (45).”

Responsibility/blame for the COVID-19 was not made explicit by either of the two newspapers during this time period. The newspapers acknowledged that the virus originated within Wuhan, China with no allocation of blame on any one group or process. Praise for China's co-operative approach and efforts to mitigate blame were apparent in the SMH:

(SMH005)—Societal—Moral Evaluation: “The Department of Foreign Affairs [Australian government department] said a Qantas plane had left Sydney yesterday for Hong Kong on the first leg of an assisted departure operation for which Chinese co-operation remains essential. “We are grateful to the Chinese government for its co-operative approach to date in this matter,” a DFAT spokesman said (45).”

(SMH008)—Societal—Moral Evaluation: “The Chinese government has expressed disappointment with travel bans to and from China instigated by various countries. They need to understand that this is not an attack on China or the Chinese people. It is only a sensible extension of what China is doing internally with their own travel restrictions (46).”

#### The Middle (Weeks 5 and 7)

During the “middle” time period, the number of newspaper articles related to COVID-19 dramatically increased coincident with the exponential trend of increasing COVID-19 cases. The escalation of the pandemic set the tone for the framing with the media reinforcing the domestic risk posed by the pandemic. Table 5 displays the prominent frames and frame components during week 5 and 7. This time period represented the first COVID-19 related death in Australia and the first swath of public health strategies to prevent further COVID-19 transmission by the federal government. The measures included travel bans, 14 days self-isolation for travelers and the release of a national emergency response plan for COVID-19 (4). By this stage, the economic effects of the pandemic were apparent with turmoil in the domestic and global financial markets. The need to balance the public health response with the economy was also discussed during this period. For example:

(AUS034) Societal—Problem Definition: “A second risk for investors is that governments in the West choose to sacrifice economic growth to try to slow the spread of the virus, as China did….Widespread school and office closures and quarantining of cities may slow the outbreak a little, but would deepen the economic damage. Japan is already going this way (47).”

(SMH21) Societal—Problem Definition: “Economic growth in NSW could slump to the lowest rate since the recession of the early 1990s as key industries in the state struggle with the effects of the coronavirus outbreak and summer bushfires (48).”

**Table 5:** Week 5 and 7, framing of COVID-19 (*n* = 55).

	**Causal attribution**	**moral evaluation**	**Problem definition**	**Treatment recommendation**
Medical	Cruise ship confines Droplets coughing/sneezing High infection rates (R0) Person to person/close contact	Authorities and researchers' mistrust of foreign countries epidemiological data Poor hygiene practices—surfaces	Increasing cases Comparisons to SARS/MERS/Colds/influenza Deaths Predictions of spread difficult Demographics—vulnerable groups Potential healthcare overcapacity Weather- winter No vaccine	Infection and recovery = resistance Seek medical advice flu-like symptoms Slow the spread Quarantine Preparations
Behavioral	Individuals unsanitary actions	Individuals putting the community at risk	Individuals not taking pandemic seriously	Self-prescribed self-isolation
Societal	Spread of coronavirus Pandemic Coronavirus outbreak/epidemic	Gov. making good decisions Markets acting indifferently to circumstance Panic selling on markets Opportunistic price gouging	Economic Impacts Border closures Supply lines disrupted Job losses Global financial market Unknown timing of impacts Education markets Tourism Recession Opportunity investment School closures Already weak economy Economic growth vs. public health Climate/environment positives Sports Canceled games TV commitments Post-Covid-19 disruption Become an established pathogen Humanitarian—asylum seekers, migration ceased	Cancel flights Screening Travel advice Downgrade economic forecasts Evacuations Gov. imposed quarantine Control spread of Coronavirus Businesses call for aid Government stimulus aid request Less reliance on China more self-reliant Improve consumer confidence Government financial packages Passenger screening Develop vaccine Invest in public health tools Travel advise to other countries Review strategy effectiveness Opportunistic investment

*Shading of cells represent relative frequency of themes (white = uncommon, light gray = few instances, dark gray = common, black = very frequent occurrence)*.

The positive and negative longer-term outlooks of the pandemic were also being discussed in relation to opportunistic investment and climate/environment. For example:

(SMH026) Societal—Problem Definition: “Australian iron ore producers are set to benefit in the fallout from coronavirus as China will ultimately seek to stimulate its economy by investing in infrastructure (49).”

(SMH030) Societal—Problem Definition: “This brings us back to climate. If our economy is severely disrupted, the government will argue we cannot afford any more risks to jobs. It may even argue our coal exports are crucial to getting the global economy going again (50).”

The medical frame remained a secondary frame. Here, problem definitions were associated with the increase in epidemiological data available and thus the confirmed cases, origin, transmission mechanisms and risk profiles of various demographics were being discussed. As per the “beginning” weeks the emphasis remained on the number of cases illustrating the rapidly changing situation in Australia. For example:

(AUS023) Medical—Problem Definition: “It is understood Australians will have to pass a coronavirus check before being taken off the ship and vulnerable elderly will be the first to be brought home. Of the 200 Australians aboard, 16 have tested positive to the virus. So far 355 people have tested positive, after 70 new cases were found on Saturday (51).”

(SMH020) Medical—Problem Definition: “Two people in close contact with a confirmed case of coronavirus could be the first person-to-person transmissions in Australia. A man in his 40 s was diagnosed with COVID-19 following recent travel from Iran, NSW Health advised yesterday. The man isolated himself as soon as he became ill (52).”

Treatment recommendations focused on public health initiatives as actioned by the federal government in tandem with state governments. Within the societal frame, this largely drew on travel bans, businesses acting toward their financial interests and occupational health and safety and calls for financial aid from all sectors of the community. For example:

(SMH023) Societal—Treatment Recommendation: “The Morrison government put a travel ban on people coming from Iran as of yesterday because of the country's high death rate from coronavirus (53).”

(AUS035) Societal—Treatment Recommendation: “In a move to protect cashflows, companies are expected to delay paying their bills as the Reserve Bank warns the coronavirus outbreak poses a material risk to the national economy, which has had a 28-years run without a recession. Already some of Australia's biggest companies, such as construction giant CIMIC, have been using supplier “payday lending”-like schemes to blow out payment times, adding further pressure to supplier cashflows (54).”

(SMH029) Societal—Treatment Recommendation: “To have an impact Dr. Oliver said federal government stimulus measures would need to be worth “at least” $10 billion, and probably around $20 billion, with the latter figure equal to about 1% of national gross domestic product (55).”

Within a medical context, the discussion on treatment focused on evidence-based processing of patients and epidemiological flattening of the curve through public health measures including quarantine. Of particular note, the time period also included the first indirect mentions of “herd immunity” as a possible outcome of infection, and loosely implied this as a potential treatment. The time period also made mention of the potential for vaccine development, and the need for preparation of the healthcare system given its limited critical care capacity. For example:

(AUS028) Societal—Treatment Recommendation: “But given how new COVID-19 is, there is no comparable scheme or vaccine that will help people become more resistant to the virus, although evidence suggests people who have been infected will be more immune in the future. Mr. Senanyake [researcher] said research showed COVID-19 wasn't mutating much, and that might help people build a resistance to it. “We suspect that in the short term if you get infected with COVID-19, you will be immune,” he said (56).”

(SMH012) Societal—Treatment Recommendation: “We must anticipate a spread of infections from now and must build medical systems and so on to focus efforts to prevent people from becoming gravely ill or dying (57).”

Moral Evaluations increased in prominence during the “middle” period. As per the “beginning” period these remained predominantly within the Societal Frame with more positive feedback for the government's response and some conflicting sentiment on financial markets behaving irrationally or indifferently. This also marked the first mention of opportunistic price gouging by businesses as demand outstripped supply for certain items, although occurrences of this complaint were infrequent. For example:

(AUS029) Societal—Moral Evaluation: “The coronavirus is becoming a most challenging national and international pandemic. The Morrison government has not put a foot wrong. Health Minister Greg Hunt and Chief Medical Officer Brendan Murphy have been superb for keeping all Australians in the loop (58).”

(SMH011) Societal—Moral Evaluation: “Pharmacy Guild Victorian president Anthony Tassone referred the issue to the Australian Competition & Consumer Commission last week, accusing Livingstone Pty Ltd. of “being opportunistic in significantly increasing the prices of their goods during a public health scare to maximize profit and price-gouge customers (59).”

Explicit framing of blame/responsibility was muted for this time period as per the “beginning” period although may be considered implicit in references to the origin of the virus.

(SMH018) Societal—Treatment Recommendation: “CSL, one of the world's largest biotechnology companies, has joined the global effort to combat the virus, lending its technical expertise and Seqirus vaccine to bolster the University of Queensland's efforts to develop an inoculation for Coronavirus (COVID-19). Coincidentally, CSL's existing Chinese facility and its 600 staff are situated in the Hubei Province at the epicenter of the epidemic (60).”

#### The End (Weeks 9 and 11)

By the “end” of the study period, several significant government guidelines aiming to flatten the epidemiological curve and provide support to citizens and businesses were in effect. These included several financial packages related to Medicare (Australian global healthcare system), JobKeeper (a new Australian government financial package aimed at maintaining employment), and income support (in the form of one-off payments to qualifying citizens). In terms of public health guidelines, limits to non-essential gatherings and restrictions on travel and aged care facilities were in place. At this point, the behavior of individuals was highlighted in regard to panic buying as the reality of the pandemic began to dawn on the population. Table 6 displays the frames and frame components most prevalent in the final weeks of the study. Framing of the COVID-19 pandemic remained firmly within the societal frame. The medical frame was secondary but of note was the rise of the Behavioral frame for the first time in the study period.

**Table 6:** Week 9 and 11, framing of COVID-19 (*n* = 234).

	**Causal attribution**	**Moral evaluation**	**Problem definition**	**Treatment recommendation**
Medical	High infection rates (R0) Person to person/close contact Weather—winter Symptoms after close contact	Triage—who gets a ventilator Elderly side-lined Dangerous situation given poor testing Healthcare workers not social distancing Fear of healthcare system overwhelmed	No vaccine developed, in testing Trials slow—ethical issues Cases/deaths increasing/reducing Demographics—vulnerable groups Impact on frontline workers—doctors/nurses Children low infection/symptoms Disaster advanced too late to stop Potential healthcare overcapacity resources finite Transmission risk—surfaces Disruptions elective surgery delay other clinical trials Flattening of curve working	Social isolation Flatten the curve Slow the spread Prepare for worse Total Isolation Pre-emptive school closures Assembly ban Hand sanitizer Testing if individual has come from oversees, fever, acute respiratory syndrome Sanitations—was hands, don't handle cash Financial aid—health services Check temperature Identify nature of super spreaders
Behavioral	Sharing kitchen utensils asymptomatic individuals not self-isolating	Disgraceful behavior in shops Hypocritical actions Self-aggrandizing Irrational actions Selfishness Flouting social isolation—beach Judgements on moralizers Armchair experts	Close contact risks ignored Self isolation for the good of the community Missing out on life Misleading information being circulated—social media Mental Health impacts of social isolation—domestic violence	Legal enforcement Random checks on individuals Fining individuals Retain social contact zoom Exercise keep mentally fit
Societal	Spread of coronavirus Pandemic Coronavirus outbreak/epidemic Virus spread from Wuhan	Slow response Panic buying Process ineffective/unprepared Gov. making good decisions/sensible Markets irrational China slow to act Poor conditions wet markets Political opportunism Calls for aid from big business obscene Careless decision making hurting business Poor media response	Economic Impacts Supply lines disrupted Job losses Global financial market Education markets Recession Opportunity investment School closures Businesses/household rents Post COVID-19 Virus timing unknown Change life as we know it—new normal Sports Canceled games TV commitments	Wage freeze, pay cuts, job losses Postponement of events Government stimulus Ban cruise ships in Aus. ports Public health act police powers Purchase limits in shops Private sectors consulting biosecurity experts Sports play without crowds, postpone 1.5m distancing, no physical contact Keep Schools open 14-day isolation ban on mass public gatherings

*Shading of cells represent relative frequency of themes (white = uncommon, light gray = few instances, dark gray = common, black = very frequent occurrence)*.

The Problem definition and treatment frame components for both the medical and the societal frames remained as the prominent components reinforcing the “the problem at hand”. The problem largely concerned the same economic and disruptive issues as per previous periods for the societal frame.

(AUS100) Societal—Problem Definition: “Sports broadcasters and administrators are scrambling to check the fine print of sports rights contracts worth hundreds of millions of dollars amid the threat of top football codes and the Olympics being suspended or canceled (61).”

(SMH129) Societal—Problem Definition: “Smaller operators have received protections from insolvent trading due to the coronavirus economic slowdown, but experts warn thousands will be facing long payment terms and unpaid invoices with little option for recourse (62).”

Within the medical frame the problem definition concentrated on the potential over capacity of healthcare services as per foreign states highlighting the problem as it was yet to arrive. As per previous periods, the number of confirmed cases ranked high as an indicator of the problem signifying the rapid change of the situation.

(AUS046) Medical—Problem Definition: “At the start of last week, Australia had reported 63 cases of COVID-19, 10 of them involving passengers taken off the Diamond Princess cruise ship in Japan. On Sunday, the total had climbed to 298, headed by NSW with 134 cases and Victoria with 57. NSW reported a spike of 22 new infections in a day, while Queensland had 26 additional cases over the weekend (63).”

Treatment recommendations for the frames were based on the immediate economic impacts for the societal frame, following the previous period's results on treatment. Although the economic disruption and call for aid dominated the discussion, as with the previous time periods, more reactive treatments to the immediate large-scale societal issues were being sought.

(AUS051) Societal—Treatment Recommendation: “Employers including [supermarket and hardware stores] IGA, FoodWorks and Miter 10 have called for a 1-year wage freeze to be imposed on retail workers, warning the coronavirus crisis could persist for at least 12 months (64).”

(AUS068) Societal—Treatment Recommendation: “Woolworths [supermarket] has suspended its online shopping in response to the shortages, while all supermarkets are limiting purchases of goods including toilet paper, hand sanitizer and non-perishable items such as pasta and rice to limit hoarding (65).”

The medical frame tended to focus on longer term preventative measures such as flattening the curve, pre-emptive school shutdowns and more robust testing regimes. This was more pronounced than the previous period and indicated the medical community's acceptance of the long-term effects of COVID-19 and it's potential to overwhelm the healthcare system as was occurring in Italy and Spain.

(AUS059) Medical—Treatment Recommendation: “We believe it is vitally important that we take swift action to reduce the number of people in close contact with others, for sustained periods of time, in order to slow the rate of COVID-19 infection,” Ms. Lloyd-Hurwitz said (66).”

(SMH083) Medical—Treatment Recommendation: “Our healthcare capacity is finite. As a past president of the Australasian College for Emergency Medicine, Simon Judkins, tweeted: “Part of the pandemic plan is “hospitals opening their surge capacity”. Now, I don't want to alarm anyone, but there is no surge capacity… we are full every day.” Experts are working to increase that surge capacity, but this involves extraordinary measures. We can help them by slowing the surge (67).”

Negative sentiment of individuals was expressed most definitively during the “end” weeks of the study period. These moral evaluations coincided with news reports of panic buying and the first signs of epidemiological flattening of the curve. Derision of hypocritical actions as well as poor social distancing behavior were most prevalent in the moral evaluations per the Behavioral Frame.

(AUS062) Behavioral—Moral Evaluation: “Recall that memorable Twitter post of some expert standing at the microphone lecturing all of us on the dos-and-don'ts of living with a virus that is as capricious as it is dangerous. She was advising us not to put our hands anywhere near our face. Then, in order to turn the page in her notes, she stuck a finger in her mouth to wet it (68).”

(AUS107) Behavioral—Moral Evaluation: “Life's a beach Victorian Liberal MP Tim Smith didn't hold back after seeing footage of the covidiots at Point Addis in Anglesea, Victoria, this weekend: “I wonder if these dickheads realize they are pushing the state government into locking all of us in our houses, literally like home detention, because these tools wanted a day at the beach. Wake up—treat this disease seriously … (69).”

Moral evaluations of society tended to focus on the irrationality of sellers on financial markets and opportunism of politicians

(SMH054) Societal—Moral Evaluation: “Our modeling also suggests that if there are no further major surprises about the severity of the pandemic and markets respond in an orderly fashion, then the Australian economy would take the better part of a decade to get close to its pre-COVID-19 trajectory,” KPMG says. “If the pandemic is more acute and long-lasting and businesses and consumers lose confidence, then markets could be disrupted by irrational behavior and the economic consequences could be more severe (70).”

(AUS056) Societal—Moral Evaluation: “We are just at the beginning of a pandemic and it is not time to play petty politics. If Labor has concerns it should be taking them up privately with the government, not using the virus as another opportunity to carp (58).”

Evaluations of blame/responsibility within the final “end” time period were rare as per the previous “beginning” and “middle” time periods. A retrospective word search to confirm the framing and thematic coding of “blame,” “mistake,” “fault,” “responsibility,” and their associated synonyms yielded no additional results as related to the COVID-19 pandemic. The moral evaluation frame component provided the first explicit examples of responsibility for the COVID-19 pandemic although these were exceptionally rare:

(AUS056) Societal—Moral Evaluation: “The coronavirus has been difficult to treat if only because the Chinese government refused for 2 months to advise the world that the virus was deadly and spreading quickly (58).”

(SMH022) Societal—Moral Evaluation: “We also need to remember that had the Chinese government listened to the doctor, now deceased, who warned them of a new and dangerous illness, instead of imprisoning and persecuting him, this virus might have been contained. The PM should not be persuaded that the economy and Chinese interests override our nation's health. This is not a time for appeasement (71).”

### The Australian vs. the Sydney Morning Herald

There were several differences in relation to the reporting between the two newspapers. Firstly, both the Australian and the SMH newspapers began publication of the COVID-19 in late January 2020. The newspapers maintained a consistent publication rate per day until the week of the 24th February 2020. This week coincided with a dramatic increase in case numbers and also the first death in Australia (Sun, 1 March). Thereafter, the number of publications increased with the Australian consistently publishing more articles on the subject.

From the onset of COVID-19 reporting the reporting styles differed between the two newspapers. As part of the coding, Author TT allocated the newspapers to content categories such as general news or finance/business. This was a straightforward process for the SMH, where the difference between the articles was fairly well-demarcated with news reports generally objectively reporting the observations of the day and opinion pieces clearly signaling a guest author and their credentials. However, in the Australian this was not as well-defined, with many otherwise objective news reports carrying with them some element of opinion which framed the narrative. No meaningful differences were discerned between the newspapers in apportioning blame as related to the primary question of the study.

Both papers published a broad range of topics, however the SMH appeared to publish heavily on the disruption to sporting events as related to rugby league, which is predominantly based in the East Coast of Australia, where the SMH is published. The Australian published on these topics but not with the frequency, most likely indicative of their more national readership demographic.

Moralizing within the newspapers was most evident in the SMH through readers' letters and guest opinion pieces. In the Australian this was also the case however with a certain inclusion of opinion in many general news articles. For example, in an article by the Australian about panic buying, the article objectively described the effects of panic buying at particular stores in Sydney and quotations from store representatives about the disruption. However, toward the end of the document a quotation from a behavioral economist was inserted generalizing a laid-back Australian attitude as being detrimental to disaster preparedness:

(AUS047) Societal—Moral Evaluation: David Savage, an associate professor of behavioral economics at the Newcastle Business School, said Australians had a tendency to react too casually to disasters and needed to prepare more responsibly. “Australians generally don't have the disaster plans, they don't have good survival plans,” Mr. Savage told ABC News (72).

This contrasted with the SMH which typically reserved moralizing of a situation to reader's letters or opinion pieces and clearly labeled as such. For example:

(SMH022) Societal—Moral Evaluation: “His backside still smoldering from his holiday/bushfires/climate-change/sports rorts debacles, it appears our PM, now thrown into the COVID-19 melting pot, has been spurred into action. Watching Scott Morrison's COVID-19 brochure-brandishing performances reminded me of a World War II British Army instruction manual about how to react in UXB (unexplored bomb) incidents. The instruction said, “in the event of seeing a UXB officer running, try to keep up.” Bill Leigh, West Pennant Hills (71).”

The overall reporting by the newspapers was objective and roughly equivalent between the two newspapers. The use of harsher language was more apparent in the Australian compared to the SMH, this was highlighted by the very occasional selective use of colorful language.

(AUS107) Behavioral—Moral Evaluation: “Life's a beach Victorian Liberal MP Tim Smith didn't hold back after seeing footage of the covidiots at Point Addis in Anglesea, Victoria, this weekend: “I wonder if these dickheads realize they are pushing the state government into locking all of us in our houses, literally like home detention, because these tools wanted a day at the beach. Wake up—treat this disease seriously … (69)”

(AUS108) Societal—Problem Definition: “[Regarding the filming of a cooking television show] Essential services? Eat your heart out, intensive care nurses! Another POO [Plate of Origin—television show] staffer texted Seven would be “lucky to get another week” of filming in. But the staffer is blunt about the network's attitude to pressing ahead with POO: “I don't think they give a shit unfortunately. Just trying to squeeze every bit of life out of something that is already dead.” Ouch.

## Discussion

The study aimed to define where the media placed responsibility for the COVID-19 pandemic, be it explicit or not, and this discussion will concentrate on the key themes identified by the framing analysis, including the apportioning of blame. This discussion will also describe the evolution of framing of the COVID-19 pandemic by the media over the course of the study time period from when the COVID-19 pandemic was established as a potential disruptive event to the height of the epidemiological curve.

### The Rise of the Wuhan Virus

Australian printed media were very slow to engage in discussion of the emerging COVID-19 pandemic. Despite the first COVID-19 case appearing as early as 1 December 2019 (30) ADDIN EN.CITE (30, 31) and whilst online reporting of an emerging influenza-type virus in the media began appearing 6 January 2020 (73), printed media picked up the story 3 weeks' later on 20 January 2020 (SMH001). For context, there were at this stage already 204 confirmed cases and one death globally (8). Given the importance of the media in providing timely information to individuals (74), the lateness of printed media in particular to engage with the emerging pandemic and distinct lack of blame for the pandemic represents an interesting question into how the media might have interpreted the threat of COVID-19. Given recent pandemics including the H1N1 pandemic also suffered from a lack of reporting in mainstream media relative to their increasing transmission (75), an underestimation to the weighting of risk of COVID-19 by the media is implied.

### Getting to Grips With the Issue

Economic risks associated with the COVID-19 pandemic were emphasized to a high degree with results showing that the overwhelming response by the media across the time periods to the escalating pandemic was related to economic disruption. As per the analysis, this was framed as a societal problem definition, with the themes of financial market turmoil and disruption to businesses pervasive throughout the study. Over the study period, the examples of social issues related to economic impacts were increased but, as might be expected, moved from a prospective view on the potential effects such as recession to a retrospective view of the cost to business and tax payers for government financial aid packages.

As with previous pandemics the highlighting of risks can serve to increase public concern and increase engagement in precautionary measures (74) and in some cases can result in irrational behaviors (76). The media highlighting discourse into the perceived risks of the COVID-19 confirms previous media studies related to Ebola (77), Zika (76), and SARS (74). However, in these examples the nature of the perceived risk was more aligned with personal health risk and therefore mortality through transmission as opposed to the fiscal risk of COVID-19 through business disruption.

### The Antidote

Generally, the economic impacts of the pandemic were followed by recommendations a fiscal nature such as requests for aid, subsidies and stimulus packages. However, the dominant treatment or solution to the prevailing conditions were initially of a public health nature. At the outset, a public health response was highlighted with examples emphasizing border closures and social distancing measures but, as time progressed, quickly turned to financial solutions through economic stimulus and aid packages as called for by business and actioned by the government. Early on it was noted that a balanced public health response with respect to the economy should be actioned. These examples highlight the objective inclinations of the media to focus on “action” and “consequence” to construct their narratives as opposed to more subjective or emotional responses to the pandemic, replicating previous studies results in defining the approach of printed media to disease outbreaks (32, 75).

### Pointing Fingers

Moral evaluations over the study period were varied in the tone of the response and specificity of blame was often opaque. In the beginning period, moral evaluations were largely directed at the government and to elements of process. This was largely praise related to the government's fast response, which was highlighted as a contrast to the recent bushfire disasters where the government were seen as slow and ineffective in their response (78). Some negative sentiment surrounding confusion and disorder of process in relation to evacuation of Australian residents from Wuhan, China, were present but infrequent. Over time, further moral evaluations of the community, that might imply responsibility, became apparent but were infrequent, vague and indirect. Panic related to irrational behaviors on the financial markets was highlighted as a societal issue and dominated the moral evaluations at this point. At the “end” of the study period, which coincided with passing the peak of the epidemiological curve and therefore a slow-down in COVID case frequency, moral evaluations had increased in frequency considerably and were now more emotionally charged, including direct denigrations of poor behavior of citizens panic buying, of the triage process and who was deserving of ventilators. The bulk of the moral response was still concerned with societal issues related to financial markets. The federal government still garnered considerable praise for their actions.

Explicit blame for the COVID-19 pandemic was sparse, indirect and infrequent during the study period. While the virus was frequently depicted as originating from China, it was only at the end of the study period that direct criticism of the Chinese pandemic response was found, and even then, instances of these were very few. The disinclination to frame responsibility for the pandemic was also made apparent in some SMH articles in the beginning period, with later representations of blame primarily exemplified within readers' letters. This contrasts to the media sentiment at the time of writing (post-study period) where a significant media effort to apportion blame to China for its slow response is underway (79).

Our study has shown that, rather than allocating responsibility, the Australian media have remained objective in their reporting following common “action” and “consequence” tropes as identified in previous studies (32). Allocating blame as a method of making sense of a crisis and allaying fears is well-known (20, 34, 80). Blame is usually apportioned to geographically distant groups with the mechanisms for assigning blame often including *othering*, and is commonly used as a tool by the media as a form of reassurance in the face of crisis (80, 81). The relative absence of immediate blame during the study period represents a divergence from previous epidemics/pandemics (20). One explanation could be that, whilst othering and therefore allocation of blame to an external “actor” is a method of reassurance, the fact that blame was almost absent until the height of the epidemiological curve had passed (i.e., higher risk was over), implies that print media did not accept the risk was high enough within Australia to merit it, therefore blame was never explicit. Further, reported examples of blame occurred on the other side of the epidemiological curve and therefore when perceived risk was reduced. This is more in tune with a retrospective accounting of the pandemic and more aligned with a government inquiry or investigation rather than use as a coping mechanism. The reluctance of the media to portray responsibility is potentially justified by the nature of the risk as it existed in Australia with low case numbers and a low case fatality rates, relative to other countries such as Italy and Spain where the health effects and associated public health response were a lot more severe (8). Another explanation could be that the blame was thought to be so obvious as not worthy of further comment, supported by the fact that the geographical origin of the virus was often reported. Alternatively, in the study period government intervention and public health directives were updating almost daily, and therefore it is possible the print media were focused on the rapidly changing societal environment and its short- and longer-term implications rather than the apportionment of blame. This is supported by the finding of greatest focus throughout all time points on the societal -problem and treatment frames, and previous literature suggesting timing and messaging by the media during times of crisis represents an important medium to manage public awareness, expectations and ultimately behavior in light of a pandemic (74).

## Strengths and Limitations

The volume of articles published within the study time frame using the search terms was simply overwhelming for hand-coded framing and thematic analysis. For this reason, many other state-wide newspapers and interstitial time periods were excluded from the study to reduce sample size. Other forms of media such as social media, blogs and television were also excluded based on the same premise. These forms of media were also excluded due to the inability of being able to search and retrieve from them systematically. For these reasons representation of examples may be incomplete in answering where the media placed responsibility for the COVID-19 pandemic. The truncated study period relative to the current progress of COVID-19 also represents a limitation and was based on the data available at the time of commencement of the study. This is noted as a common limitation in the study of pandemics and given the unknown length of many social distancing guidelines may represent a “lingering crisis” (75, 82). At the time of writing, some significant geo-political inquiries are being sought by various governments into the handling of the COVID-19 pandemic which would have potentially added further examples of how the media apportioned blame/responsibility for the pandemic (79). Overall, our study adds to the existing literature in describing shortfalls and strengths in how the media responded to framed responsibility for the COVID-19 pandemic.

## Conclusion

Our findings provide several insights into how the media framed responsibility for the COVID-19 pandemic of 2019/2020. The distinct lateness in publications related to COVID-19 and the lack of blame potentially represents an indication of how the media have interpreted the risk as posed by the COVID-19 pandemic in Australia. The perceived risk by the media may be justified based on confirmed cases and total deaths in Australia relative to other affected countries.

## Data Availability Statement

The original contributions presented in the study are included in the article/supplementary material, further inquiries can be directed to the corresponding author/s.

## Author Contributions

TT collected the data and undertook the data analysis. AW and ET reviewed the data analysis. TT wrote the first draft of the manuscript and all authors participated in the development and editing of the manuscript, including the final approval. All authors contributed to the design of the study and its analytical approach.

## Conflict of Interest

The authors declare that the research was conducted in the absence of any commercial or financial relationships that could be construed as a potential conflict of interest.
